# Sea Buckthorn Leaf Powders: The Impact of Cultivar and Drying Mode on Antioxidant, Phytochemical, and Chromatic Profile of Valuable Resource

**DOI:** 10.3390/molecules26164765

**Published:** 2021-08-06

**Authors:** Lina Raudone, Viktorija Puzerytė, Gabriele Vilkickyte, Aurelija Niekyte, Juozas Lanauskas, Jonas Viskelis, Pranas Viskelis

**Affiliations:** 1 Department of Pharmacognosy, Lithuanian University of Health Sciences, Sukileliu av. 13, LT-50162 Kaunas, Lithuania; 2 Laboratory of Biopharmaceutical Research, Institute of Pharmaceutical Technologies, Lithuanian University of Health Sciences, Sukileliu av. 13, LT-50162 Kaunas, Lithuania; gabriele.vilkickyte@lsmu.lt (G.V.); aurelija.niekyte@stud.lsmu.lt (A.N.); 3 Laboratory of Biochemistry and Technology, Institute of Horticulture, Lithuanian Research Centre for Agriculture and Forestry, Kauno Str. 30, LT-54333 Babtai, Kaunas District, Lithuania; viktorija.puzeryte@lammc.lt (V.P.); jonas.viskelis@lammc.lt (J.V.); pranas.viskelis@lammc.lt (P.V.); 4 Department of Horticulture Technologies, Institute of Horticulture, Lithuanian Research Centre for Agriculture and Forestry, Kauno Str. 30, LT-54333 Babtai, Kaunas District, Lithuania; juozas.lanauskas@lammc.lt

**Keywords:** sea buckthorn, phenolic compounds, triterpenic compounds, carotenoids, chlorophyll, freeze-drying, leaf powder ingredients

## Abstract

Sea buckthorn (*Hippophae rhamnoides* L. (HR)) leaf powders are the underutilized, promising resource of valuable compounds. Genotype and processing methods are key factors in the preparation of homogenous, stable, and quantified ingredients. The aim of this study was to evaluate the phenolic, triterpenic, antioxidant profiles, carotenoid and chlorophyll content, and chromatic characteristics of convection-dried and freeze-dried HR leaf powders obtained from ten different female cultivars, namely ‘Avgustinka’, ‘Botaniceskaja Liubitelskaja’, ‘Botaniceskaja’, ‘Hibrid Percika’, ‘Julia’, ‘Nivelena’, ‘Otradnaja’, ‘Podarok Sadu’, ‘Trofimovskaja’, and ‘Vorobjovskaja’. The chromatic characteristics were determined using the CIELAB scale. The phytochemical profiles were determined using HPLC-PDA (high performance liquid chromatography with photodiode array detector) analysis; spectrophotometric assays and antioxidant activities were investigated using ABTS (2,2′-Azino-bis(3-ethylbenzothiazoline-6-sulfonic acid)) and FRAP (ferric ion reducing antioxidant power) assays. The sea buckthorn leaf powders had a yellowish-green appearance. The drying mode had a significant impact on the total antioxidant activity, chlorophyll content, and chromatic characteristics of the samples; the freeze-dried samples were superior in antioxidant activity, chlorophyll, carotenoid content, and chromatic profile, compared to convection-dried leaf powder samples. The determined triterpenic and phenolic profiles strongly depend on the cultivar, and the drying technique had no impact on qualitative and quantitative composition. Catechin, epigallocatechin, procyanidin B3, ursolic acid, α-amyrin, and β-sitosterol could be used as quantitative markers in the phenolic and triterpenic profiles. The cultivars ‘Avgustinka’, ‘Nivelena’, and ‘Botaniceskaja’ were superior to other tested cultivars, with the phytochemical composition and antioxidant activity.

## 1. Introduction

In the frame of the changing climate, more attention is paid to the plants that are resistant to the environment, have ecological implications, and are important for maintaining human and animal wellness [1]. *Hippophae* L.—genus (Elaeagnaceae Juss.) consists of seven dioecious, wind-pollinated species, the most known among them is sea buckthorn, *Hippophae rhamnoides* L. (HR) [2]. The species are widely distributed in the Northern Hemisphere and have great adaptability features to various climatic and edaphic conditions. *Hippophae* plants are native to Asia and Europe, and they can be found in North America [3,4]. The species are also cultivated in plantations as an agriculture crop using diverse genetic origin cultivars (which have possessed specific traits and suitability for different climatic zones since the 1970s) [3,5]. The HR plants can withstand a great range of temperatures and they are resistant to drought. Moreover, the HR plants can easily develop a complex root system coupled to nitrogen fixation and be used for soil erosion prevention or be suitable for planting in degraded soils [3,6]. All parts of HR plants and their extracts can be used for pharmaceutical, nutraceutical, cosmetical, food, and fodder purposes, with the most well-known materials being fruits and leaves [4,5,7,8]. The traditional medicinal systems incorporate the fruits and leaves of HR in the treatment of various ailments of digestive, hepatic, and cardiovascular systems, as well as skin diseases [3,4]. In vitro and in vivo studies have confirmed the anti-inflammatory, antitumor, hepatoprotective, immunomodulatory, anti-atherogenic, anti-stress, hepatoprotective, radioprotective, tissue repair, antibacterial, antifungal, antiviral, and antioxidant activities, which are determined by the multichemical origin compounds [4,9,10,11,12,13]. Fruits and leaves contain rich profiles of carotenoids, tocopherols, amino acids, triterpenic compounds, and phenolic compounds [3,5,7,8,10,14,15,16,17,18,19,20]. The phenolic compounds determine the pharmacological effects that are associated with the antioxidant activity [10,21,22]. Carotenoid intake is associated with a reduced risk of chronic aging-related diseases [23]. Chlorophylls can suppress radical species from mitochondria and can have antiproliferative effects on cancer cells, as well as modulate the redox status [24]. HR extracts can modulate intracellular oxidative stress, prevent mitochondrial impairment, and protect neuronal cells from damage [16]. On the other hand, the multitude of different chemical origin substances acting in different modes provide synergistic or additive effects [11,25]; therefore, the comprehensive determination of phytochemical profiles could provide information necessary for the standardization of extracts. The variable phytochemical characteristics can occur depending on the genotype, female or male plant, the climatic zone of the growing area, cultivation conditions, harvesting time, post-harvest management, and extraction methodology [3,12,20,21,22]. Growing promising genotypes in plantations could ensure greater homogeneity of raw materials with defined markers for standardization [26]. Due to the specific attachments of fruits, the branches are pruned during harvesting. As the leaves are also a very promising raw material, no-waste technologies could be promoted [27]. The leaves contain similar phytochemical profiles as fruits, but have significantly higher amounts of phenolic compounds, especially hydrolysable and condensed tannins, triterpenic compounds, and flavonoids [10,19,20,21]. Flavonol isorhamnetin, in the frame of the COVID-19 pandemic situation, gained scientific attention, due to its capabilities of in vitro inhibition on the entrance of SARS-CoV-2 spike pseudo-typed virus into cells [13]. Results suggest great potential for isorhamnetin-rich materials of HR as candidates in COVID-19 management. Literature states that the phytochemical composition and antioxidant activity of HR leaves are comparable to the green tea [9]. The drying methods also have a significant impact on the quantitative profile, as well as the color of the product [28]. Color changes can be induced by various reactions occurring in the raw materials during the drying process [29]. The elevated drying temperatures can reduce the total amounts of carotenoids and phenolic compounds [30,31,32]; therefore, the evaluation of the conventional drying methods and innovative techniques, such as freeze-drying, is crucial to produce high-added value products with unaltered health properties. The color characteristics have been evaluated for the HR fruit products [32], but no data were found regarding the leaves. Furthermore, the phytochemical profile data on HR cultivars’ leaves is still scarce, especially on triterpenic compounds. This is the first report on the detailed triterpenic composition of leaf samples. The adaptability traits, together with rich phytochemical compositions and pharmacological potential, propose HR as a multifunctional plant for the promotion of no-waste technologies, including the better exploitation of plant material resources and growth of sustainable agriculture. The aim of this study was to evaluate the phenolic, triterpenic, antioxidant, and chromatic profiles, as well as the carotenoid and chlorophyll content of convection-dried (SD) and freeze-dried (FD) HR leaf powders obtained from ten different cultivars. To the best of our knowledge, this is the first comparative report on the processed leaf powders of ten collectional cultivars.

## 2. Results and Discussion

### 2.1. Evaluation of Chromatic Parameters in the Freeze-Dried and Convection-Dried Leaf Powders of H. rhamnoides Cultivars

The processing stage is necessary for the plant origin materials to become stable, functional ingredients or products. Thermal processing, operating in a various regimes, is applied for the preservation of materials [33]. On the other hand, it can induce the alterations in composition, as well as in color. Color is a significant quality trait for a product or ingredient linked with visual appeal, consumers’ expectations and demands, intrinsic quality potential, safety, and stability [30,33,34]. The determination of color parameters can predict quality changes and aid in the standardization procedures requisite for the stable product, corresponding to purposeful quality requirements. CIELAB color parameters provide reliable, reproducible, and comparative results [34]. The applied chromatic characteristics elucidated that freeze-drying gave better color quality parameter values, with lighter, more vivid, and greener powders. Table 1 presents the data on the convection-dried and freeze-dried HR leaves, indicating the L*, a*, b*, C, and h values. The significant differences between convection-dried and freeze-dried HR powders were determined for the cultivars tested. Significant correlations were established between the different drying modes for all color values (R^2^ ranged 0.41–0.78 and R ranged 0.64–0.88). The L* value represents the lightness (the closer to 100, the lighter the color). In some cases, the degradation of phytochemical compounds can be associated with the lowered values of the L* value indicator [32,35]. The freeze-dried HR leaf powders had significantly greater (*p* < 0.05) L* values, compared to convection-dried powders (on average, 59.88 ± 0.90 and 57.76 ± 1.62, respectively). The lightest powders were obtained from the cultivar ‘Trofimovskaja’ (61.76 ± 0.01 and 60.28 ± 0.03 for the FD and CD samples, respectively). The lower L* values can be associated with the higher temperatures’ regime during the drying process [31]. The a* values represent the shift in color towards greenish (negative values) or reddish (positive values) directions. The shifts can be linked to the retention or oxidation of chlorophylls and carotenoids [31,34]. All the obtained HR sample values ranged from −8.24–−0.30. The freeze-dried HR powders were, on average, 3.5-fold more shifted toward the green scale, compared to the convection-dried HR powder samples. The powders of ‘Vorobjovskaja’ had the greatest a* values (*p* < 0.05) in both drying modes. The b* values represent the yellow and blue colors, towards positive and negative scales, respectively. Freeze-dried powders were determined with greater yellow shift (on average, b values 24.09 ± 0.86), compared to convection-dried powder samples (on average—19.71 ± 1.60).

In general, the a* and b* values together indicate the yellowish-green appearance of the HR powders. The chrome C value represents the chroma, or the vividness of the color [29,33,34,36]. The obtained c values were greater (*p* < 0.05) in the freeze-dried powder samples for all cultivars tested and correlated with the values of a and b (R = −0.60 and R = 98, respectively). Cultivars ‘Podarok Sadu’ and ‘Trofimovskaja’ had the greatest chroma values (>23) in convection-dried mode leaf powders, while in the freeze-dried mode cultivars, the greatest values were determined for ‘Nivelena’ and ‘Botaniceskaja’ (>26) (Table 1). The h value is a color-appearance parameter that is corresponding to the dominant wavelength and represents the degrees, herein obtained angle values from 90° (yellow) towards 180° (green) and up to 110°. The obtained values correspond to the overall trend, with the freeze-dried powder samples possessing a greater shift toward the green color. Cultivar ‘Vorobjovskaja’ had the greatest h values in the freeze-drying mode. The calculated ΔE values indicate the color distance between evaluated colors. Figure S4 presents the ΔE data on fresh and dried HR material. As the values of FD materials are significantly (*p* < 0.05) lower, compared to CD materials, results suggest that freeze-drying retains the color of fresh leaves greater, compared to convection-drying.

### 2.2. Content of Chrolophyll A (Cha), Chlorophyll B (Chb), and Carotenoids in the Freeze-Dried and Convection-Dried Leaf Powders of H. rhamnoides Cultivars

**Figure 1:**
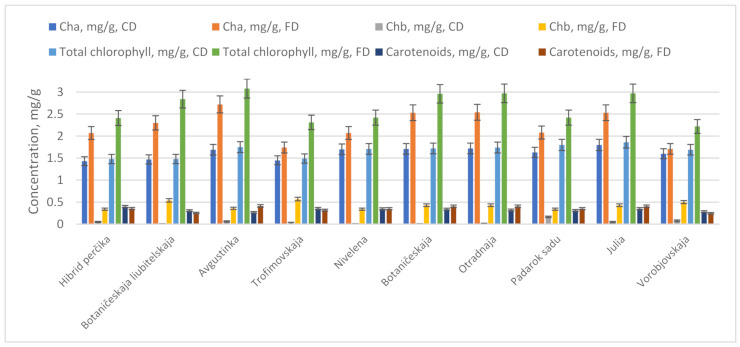
Total amounts (mg/g) of chrolophyll A (Cha), chlorophyll B (Chb), and carotenoids in convection-dried (CD) and freeze-dried (FD) HR cultivars’ leaf powders.

The chlorophyll is an important leaf pigment, providing green color and indicating the capacity of photosynthesis [28,37,38]. The drying method influenced the content of the chlorophylls detrimentally (Figure 1). Freeze-drying, compared to convection-drying, ensured greater (*p* < 0.05) retention of chlorophyll a, chlorophyll b, and the total amount of chlorophylls, on average, 1.4-fold, 19-fold, and 1.6-fold, respectively. The greatest amounts of total chlorophyll were determined in the leaf powders of the cultivars ‘Avgustinka’, ‘Julia’, ‘Otradnaja’, and ‘Botaniceskaja’ (−3.08 ± 0.22 mg/g, 2.97 ± 0.21 mg/g, 2.97 ± 0.22 mg/g, and 2.96 ± 0.20 mg/g, respectively). The impact of the drying method on the chlorophyll content varied, depending on the cultivar. However, cultivars ‘Avgustinka’ and ‘Julia’ contained the greatest amounts of chlorophyll in both drying techniques. The amounts of chlorophylls were well-correlated with all chromatic parameters (R for L*, b*, C, and h values ranged from 0.49–0.96) (R for a* value—−0.66–−0.92) and antioxidant activity (up to 0.55 and 0.61 (*p* < 0.05) with ABTS and FRAP assays, respectively). Chlorophyll b was more susceptible to drying-induced degradation, compared to chlorophyll a. Kumar et al., 2015 [38], determined that the freeze-drying method resulted in higher amounts of chlorophyll a, chlorophyll b, and the total chlorophylls, compared to thermal drying, during which auto-oxidation and various other intrinsic processes can occur. Guan et al., 2005 [30], determined that increasing the drying temperatures resulted in decreased chlorophyll content.

The amounts of total carotenoids varied significantly (*p* < 0.05) between the cultivars and drying methods (Figure 1). The greatest amounts of total carotenoids (*p* < 0.05) were determined in the freeze-dried powders of ‘Avgustinka’, ‘Botaniceskaja’, ‘Otradnaja’, ‘Julia’, and ‘Nivelena’ (0.41 ± 0.03 mg/g, 0.40 ± 0.03 mg/g, 0.40 ± 0.02 mg/g, 0.40 ± 0.02 mg/g, and 0.35 ± 0.02 mg/g, respectively), compared to convection-drying and other cultivars in both drying modes. No significant differences in total carotenoids, between convection-drying and freeze-drying, were determined for the other cultivars, namely ‘Hibrid Percika’, ‘Botaniceskaja Liubitelskaja’, ‘Trofimovskaja’, and’ Vorobjovskaja’. On average, the total amount of carotenoids in the HR leaf powders was 0.34 ± 0.01 mg/g. The best preservation of phytochemical compounds, especially the lipophilic ones, is obtained using freeze-drying techniques, as the high drying temperatures in conventional modes result in compound deterioration [39]. The amounts of determined carotenoids and chlorophylls in HR leaf powders were comparable with the amounts determined in commonly used vegetables [23,30].

### 2.3. Radical Scavenging and Reducing Activities of the Freeze-Dried and Convection-Dried Leaf Powders of H. rhamnoides Cultivars

**Figure 2:**
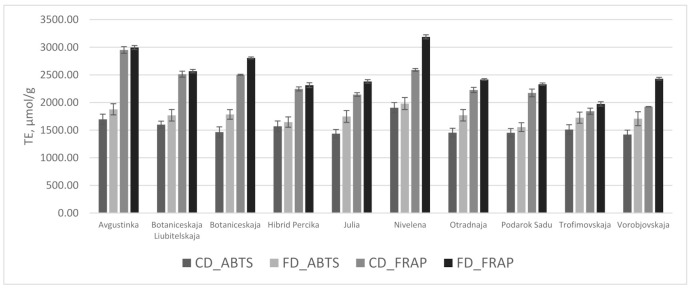
Trolox equivalent antioxidant activity values (TE, µmol/g) of convection-dried (CD) and freeze-dried (FD) HR cultivars’ leaf powders.

The determined antioxidant activity, corresponding to the radical scavenging and reducing activities of the tested HR leaf powders, is presented in Figure 2. The drying mode had an impact on the total antioxidant activity of the samples, and the antioxidant activity of the freeze-dried samples was significantly greater than convection-dried HR leaf powder samples. The leaf powder samples of ‘Avgustinka’ and ‘Nivelena’ possessed the greatest antioxidant activities in both drying modes (*p* < 0.05). The main contributors to the antioxidant activity are phenolic origin compounds. Correlational interrelationships were determined between radical scavenging, reducing activities, and the amounts of catechin, gallic acid, ellagic acid, *p*-coumaric acid, rutin, kaempferol, epigallocatechin, isorhamnetin, myricetin, isoquercitrin, and procyanidin B3; additionally, with the sum of total identified phenolic compounds, the Pearson’s correlation coefficients ranged from 0.44–0.74 (*p* < 0.05). Reducing activities were greater compared to radical scavenging in both drying modes and were well-correlated (R = 0.61, *p* < 0.05). Ellagic acid and flavan-3-ols, namely catechin and epigallocatechin gallate, possess greater reducing activities, compared to radical scavenging [40]. These compounds predominate the phenolic profile in all the cultivars tested and were determined to have 1.5-fold greater reduction activity, compared to radical scavenging. Our results are in agreement with Sne et al., 2013 [21], and Tzachristas et al., 2020 [41], as they determined significantly greater FRAP values, compared to DPPH in the leaves of HR [21,41]. In vitro antioxidant assays cannot be interpolated to the occurring effect in vivo; nevertheless, they elucidate the potential of antioxidant active compounds to express their effects in different modes of action. The selection of antioxidant activity methods should be based on their mechanisms of action, and due to the complexity of phytochemicals, at least two methods should be implemented [5,40]. However, other components present in the HR leaves, such as ascorbic acid, tocopherols, and carotenoids, contribute to the antioxidant activity, as well [20,22,30,41]. Gornas et al., 2014 [39], determined that lipophyllic antioxidants, such as carotenoids and tocopherols have been retained to a greater extent using freeze-drying technique, compared to conventional drying. Furthermore, raw materials of HR are void of ascorbic acid oxidase, which ensures the retention of ascorbic acid in dried products [10]. The HR leaf powders have great potential as a functional antioxidative ingredient in the vinification process [41]. HR leaf extracts, compared to green tea in cell cultures, increase glutathione levels, which causes intracellular redox homeostasis [9]. Ethanolic extracts also contain lipophyllic compounds that contribute to the total antioxidant activity significantly [39]. The multitude of lipophilic and hydrophilic chemical origin compounds present in the botanical matrix possess intrinsic and inter-relational antioxidant effects [10,39]; therefore, selection of the proper drying regime is a crucial step in the preparation of the antioxidant’s active ingredients.

### 2.4. Principal Component Analysis of the Freeze-Dried and Convection-Dried Leaf Powders of H. rhamnoides Cultivars

**Figure 3:**
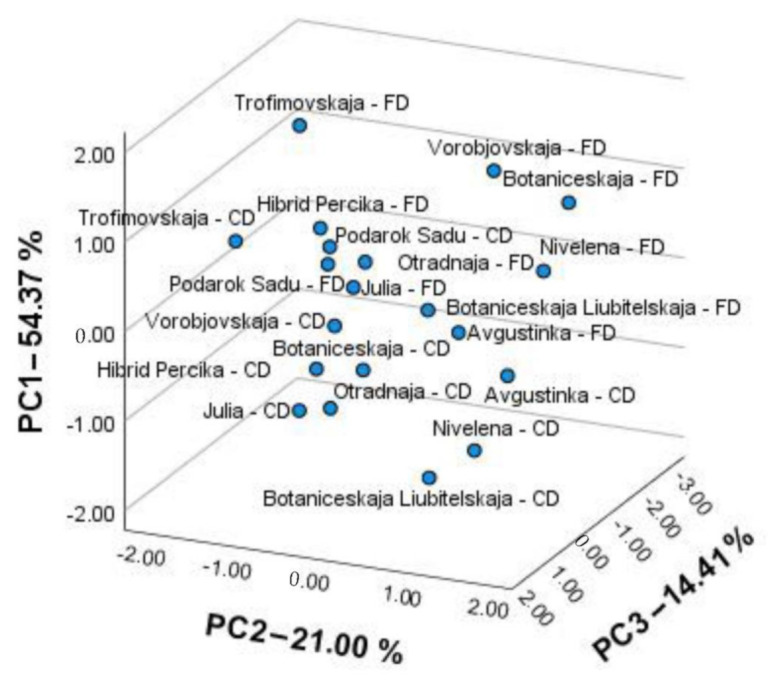
PCA score plots for antioxidant and chromatic characteristics of convection-dried (CD) and freeze-dried (FD) HR cultivars’ leaf powders.

The principal component analysis was applied to distinguish the color parameters, chlorophyll content, carotenoid content, and antioxidant activity of the convection-dried and freeze-dried HR leaf powders of different cultivars. Three principal components, PC1, PC2, and PC3 were obtained, which explained 54.37%, 21.00%, and 14.41% of the total variance, respectively. The PC1 was positively correlated with L*, b*, C, and h values, with the correlation coefficients being 0.86, 0.93, 0.94, and 0.96, respectively; additionally, chlorophyll b (0.89) and the total chlorophylls (0.66) were negatively correlated with a value (−0.95). The PC2 highly positively correlated with the FRAP and ABTS values (0.95 and 0.71, respectively). The PC3 positively correlated with the content of total carotenoids (0.95) and chlorophyll a (0.61). The score plots (Figure 3) cultivars (with different drying modes) into groups, corresponding to the drying method and cultivar.

The first group, on the top right, consisted of the freeze-dried powders of HR cultivars, namely ‘Vorobjobskaja’, ‘Botaniceskaja Liubitelskaja’, ‘Botaniceskaja’, ‘Avgustinka’, and ‘Nivelena’. They were distinguished by higher antioxidant activity and had lighter, more vivid, and closer to pure green color characteristics; additionally, they contained higher amounts of carotenoids and chlorophylls. The second group, on the bottom right, consisted of the convection-dried HR powders of three corresponding cultivars, namely ‘Avgustinka’, ‘Nivelena’, and ‘Botaniceskaja Liubitelskaja’. They possessed similar antioxidant activities but were darker, shifted toward yellow color powders, and had a lower carotenoid and chlorophyll content, compared to the first group. The third group, on the bottom left, grouped convection-dried cultivars, namely ‘Vorobjovskaja’, ‘Julia’, ‘Otradnaja’, ‘Botaniceskaja’, and ‘Hibrid Percika’. The fourth group, on the top left, grouped the cultivars ‘Podarok Sadu’ (both drying modes), ‘Hibrid Percika’, ‘Julia’, ‘Otradnaja’ (FD mode), and ‘Trofimovskaja’ (CD mode). The last two groups had significantly lower antioxidant activity, and the fourth group was characterized by its lighter, greener, and vivid colors. Cultivar ‘Trofimovskaja’ was distinguished by the greatest amounts of chlorophyll b and notable amounts of carotenoids. In summary, the freeze-dried powders of cultivars ‘Avgustinka’, ‘Botaniceskaja’, and ‘Nivelena’ were superior in antioxidant activity, carotenoid, chlorophyll content, and color characteristics. Kumar et al., 2014 [38], determined that freeze-drying retained the greatest amounts of chlorophyl, ascorbic acid, and antioxidant activity, compared to room, sun, or other thermal drying techniques [38]. Higher drying temperatures (>80 °C) induced the decay of the phenolic compounds, compared to lower drying temperatures [30]. In our study, the color parameters, radical scavenging, and reduced activities, as well as the amounts of chlorophylls and carotenoids (for certain cultivars) of the tested HR powders had greater values in the freeze-drying mode, compared to convection-thermal drying. On the other hand, the drying modes had no significant effect on the amount of identified phenolic and triterpenic compounds. This is in agreement with the results of Asofiei et al., 2019 [42], where the polyphenolic profiles were not affected by the microwave-assisted extraction. The results suggest that other compounds of the phytochemical complex with antioxidant activity can be susceptible to drying mode, such as ascorbic acid, tocopherols, carotenoids, tannins, or other unidentified compounds in the phenolic and triterpenic profiles [10].

### 2.5. Hierarchical Cluster Analysis of Phenolic and Triterpenic Compounds

**Figure 4:**
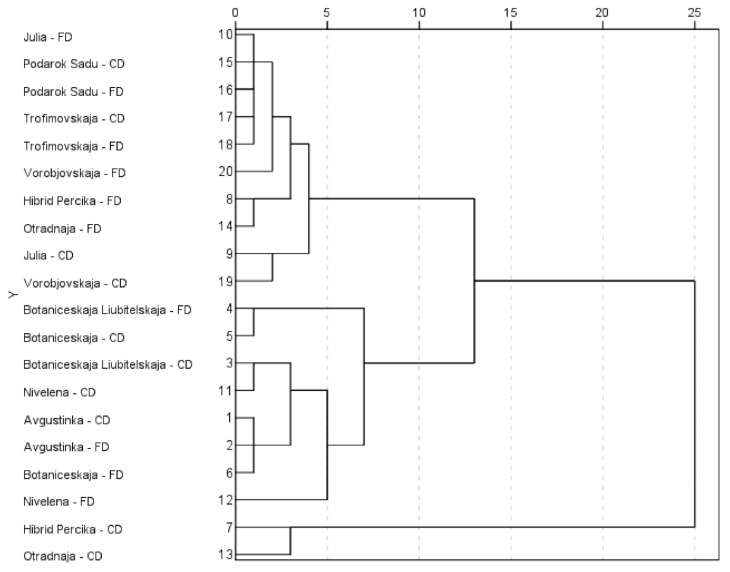
The dendrogram of hierarchical cluster analysis on phenolic and triterpenic compounds of convection-dried (CD) and freeze-dried (FD) HR cultivars’ leaf powders. 1–cluster composed of CD and FD samples of ‘Avgustinka’, ‘Botaniceskaja’, ‘Botaniceskaja Liubitelskaja’, and ‘Nivelena’; 2–cluster composed of CD samples of ‘Hibrid Percika’ and ‘Otradnaja’; 3-cluster composed of CD and FD samples of ‘Julia’, ‘Podarok Sadu’, ‘Trofimovskaja’, ‘Vorobjovskaja’, and FD samples of ‘Hibrid Percika’ and ‘Otradnaja’.

The phytochemical profiles of individual phenolic and triterpenic compounds were determined for each HR cultivar sample for both drying modes applied. Hierarchical cluster analysis was performed to the convection-dried and freeze-dried HR leaf powder samples for the mean qualities of phenolic and triterpenic compounds (Figure 4).

The cluster analysis grouped the samples into three clusters. The first cluster coupled all the convection-dried and freeze-dried samples from the cultivars ‘Avgustinka’, ‘Nivelena’, ‘Botaniceskaja’, and ‘Botaniceskaja Liubitelskaja’. They can be characterized by the greatest amounts of catechin, protocatechuic acid, ellagic acid, and the total amount of triterpenic compounds. The second cluster coupled two convection-dried samples from the cultivars ‘Hibrid Percika’ and ‘Otradnaja’. The determined amounts of the total triterpenic compounds were lower, compared to other clusters. The third cluster grouped all the convection-dried and freeze-dried samples from the cultivars, namely ‘Vorobjovskaja’, ‘Julia’, ‘Trofimovskaja’, and ‘Podarok Sadu’, as well as the freeze-dried samples from cultivars ‘Hibrid Percika’ and ‘Otradnaja’. The clustering revealed that the triterpenic and phenolic profiles strongly depend on the cultivar, as the samples from different drying methods tended to group under the cultivar. The phenolic composition has a significant genotypic and geographic-related qualitative and quantitative variability [2,9]. The principal phenolic markers characteristic of the profiles of HR leaves are ellagic acid, gallic acid, isorhamnetin, kaempferol, and quercetin derivatives [7]. The significant differences between the amounts of determined compounds in the HR powders and the different drying methods were determined only for certain compounds, and the superiority of specific drying methods was not confirmed. Therefore, the detailed discussion on phenolic and triterpenic profiles is presented on the freeze-dried HR cultivar powder samples, as they were defined with better chlorophyll, carotenoid, and chromatic characteristics (Table 2).

### 2.6. Phenolic and Triterpenic Profiles of the Freeze-Dried Leaf Powders of H. rhamnoides Cultivars

The determined phenolic profiles of 10 tested cultivars of HR consisted of flavonoids, phenolic acids, and stilbene compound resveratrol. The flavonoid complex was comprised of flavan-3-ols (catechin, epigallocatechin, epicatechin gallate, and procyanidin B3) and flavonols (rutin, isorhamnetin-3-rutinoside, isorhamnetin-3-glucoside, quercetin, kaempferol, tiliroside, isorhamnetin, myricetin, quercetin 3-*O*-6′’-acetyl-glucoside, and isoquercitrin). Flavan-3-ols were predominant compounds in the profiles of all tested cultivars, with the greatest amounts in ‘Avgustinka’, ‘Nivelena’, ‘Botaniceskaja Liubitelskaja’, and’ Botaniceskaja’ (Table 2). The total amounts of flavonol derivatives in the cultivars were in the following order: isorhamnetin derivatives > quercetin derivatives > keampferol derivatives. Their profiles were cultivar-dependent and elucidated ‘Podarok Sadu’, ‘Trofimovskaja’, and ‘Vorobjovskaja’ with the greatest amounts of isorhamnetin derivatives, up to 1088 µg/g of dry weight (dw). Chemophenetically isorhamnetin glycosides prevail over quercetin glycosides [19]. Recent findings on the isorhamnetin’s capability to bind to human angiotensin-converting enzyme 2 and prevent the SARS-CoV-2 virus from entering the cells [13] could initiate further research on the valorization of HR leaves for the production of isorhamnetin. The leaf powders of ‘Otradnaja’ and ‘Podarok Sadu’ were determined with the greatest amounts of kaempferol derivatives. Quercetin glycosides predominated in ‘Avgustinka’ and ‘Botaniceskaja Liubitelskaja’ cultivars. Ciesarowa et al., 2020 [10], in the HR leaf profiles, determined rutin and hyperoside as the predominant quercetin derivatives, whereas in our study hyperoside was quantified only in traces (data not shown). Overall, the leaf powders of ‘Avgustinka’, ‘Nivelena’, and ‘Botaniceskaja Liubitelskaja’ cultivars were significantly distinguished by the greatest amounts of total identified flavonoid derivatives, on average, 111.78 mg/g dw. The qualitative profile is in agreement with the literature data, indicating rutin, epigallocatechin, and catechin as flavonoid profile markers [7,8,19,20,43,44]. The profile of specific isorhamnetin, kaempferol, and quercetin derivatives is genotype- and habitat-dependent and can be applied to chemophenetic and authenticity studies [19].

The total amounts of identified phenolic acids ranged from 4511.40 µg/g to 6150.70 µg/g in the cultivars ‘Trofimovskaja’ and ‘Avgustinka’, respectively. The profiles were predominated by the protocatechuic, ellagic, and gallic acids in all the tested cultivars (up to 3405.10 ± 117.65 µg/g, 2157.40 ± 74.43 µg/g, and 565.90 ± 19.30 µg/g, respectively), while ferulic, *p*-coumaric, and caffeic acids were the minor compounds (Table 2). Asofiei et al., 2019 [42], determined gallic acid as a predominant compound in various modes of extraction with different parameters. The greatest (*p* < 0.05) quantitative phenolic acid profiles were determined for the leaf powders of the cultivars ‘Avgustinka’ and ‘Botaniceskaja Liubitelskaja’ and corresponded to the cultivars with the greatest flavonoid profiles. On the other hand, the amounts of phenolic acids in the leaf powders of cultivar ‘Julia’ were comparable with the total amounts of 5421.20 µg/g. Zadernowski et al., 2005 [45], in the fruit samples of ‘Nivelena’, ‘Otradnaja’, ‘Podarok Sadu’, and ‘Trofimovskaja’ quantified 1135–1868 µg/g of phenolic acids. Fruits samples were predominated by gallic and salicylic acids. Other identified phenolic acids conform to the genotype and are in agreement with the components identified in our study. Sytarova et al., 2020 [20], in the HR leaf samples, additionally determined notable amounts of chlorogenic and neochlorogenic acids; however, leaf samples of our tested cultivars were devoid of these compounds. Studies suggest that leaves contain richer fractions of phenolic acids, and individual qualitative and quantitative profiles are genotype- and habitat-dependent [3,10,20,45].

The greatest amounts of resveratrol (above 100 µg/g) were determined in the leaf powders of the cultivars ‘Otradnaja’ and ‘Podarok Sadu’. Leaves of HR genotypes, cultivated in Velke Ripnany, only contained up to 7.9 µg/g of resveratrol [20]. Ghendov-Mosanu et al., 2020 [46], determined about 100 µg/g of resveratrol in the fruit extracts. The amounts of resveratrol in the leaves of HR are comparable with the amounts determined in well-known sources, such as peanuts, red wines, or itadori materials [47].

The correlational analysis revealed strong interrelationships (R = 0.38–0.66 and *p* < 0.05) between reducing activities and phenolic compounds, namely catechin, gallic acid, ellagic acid, *p*-coumaric acid, rutin, isorhamnetin-3-rutinoside, kaempferol, epigallocatechin, isoquercitrin, and procyanidin B3. Radical scavenging activities were correlated only to the individual amounts of isorhamnetin and myricetin, 0.53 and 0.71, respectively (*p* < 0.01). The correlations between the chromatic characteristics and individual phenolic compounds were also established. The amounts of catechin, gallic acid, ellagic acid, quercetin, kaempferol, epigallocatechin, and procyanidin B3 were positively correlated with chromatic parameters b and c (R = 0.41–0.83 and *p* < 0.05). On the other hand, the amounts of caftaric acid and isorhamnetin-3-rutinoside negatively correlated to parameters b and c (R = −0.43–−0.75 and *p* < 0.05).

The determined triterpenic profile was comprised of triterpenoid acids (maslinic, oleonolic, ursolic, corosolic, and betulinic), triterpene alcohols (erythrodiol, uvaol, lupeol, β-amyrin, and α-amyrin), neutral triterpenes (botulin and friedelin), and phytosterol—β-sitosterol (Table. 2). The predominant triterpenic compounds in the profiles occurred in the following order: α-amyrin > ursolic acid > β-sitosterol > corosolic acid. The content of α-amyrin comprised of, on average, about 24% of all identified triterpenic compounds and ranged from 225.63 ± 7.53 µg/g (‘Trofimovskaja’) to 972.84 ± 33.40 µg/g (‘Avgustinka’). The amounts of ursolic acid ranged from 221.53 ± 7.39 µg/g (‘Otradnaja’) to 657.45 ± 22.47 µg/g (‘Avgustinka’) and constituted up to 23% of the total triterpenic compounds. The amounts of β-sitosterol in the leaf powders of HP cultivars corresponded to the quantitative pattern of ursolic acid, with the greatest amounts in ‘Avgustinka’ and ‘Voroblevskaja’ (373.81 ± 12.65 µg/g and 283.84 ± 9.54 µg/g, respectively) (Table 2). Kukin et al., 2017 [27], determined β-sitosterol as the predominant compound in the profile of triterpenoids and sterols. The leaf powders of cultivar ‘Julia’ were distinguished by the greatest (*p* < 0.05) amounts of corosolic acid—263.05 ± 8.82 µg/g. The greatest total amounts of identified triterpenic compounds (*p* < 0.05) were determined for the leaf powders of cultivars ‘Avgustinka’, ‘Nivelena’, and ‘Vorobjevskaja’ (3604.86 µg/g, 2584.47 µg/g, and 2599.71 µg/g, respectively). Individual amounts of triterpenic compounds, correlated only with reducing activity, indicated the highest coefficients for maslinic acid, α-amyrin, and β-amyrin (R= 0.70, 0.60, and 0.74, respectively). Furthermore, all triterpenic compounds (except maslinic acid, betulinic acid, erythrodiol, and uvaol) negatively correlated with the chromatic parameter L, (R = −0.35–−0.65 and *p* < 0.05) indicating their impact on the lightness of the powders. Certain triterpenic compounds were quantified in the fruit materials, with ursolic, oleanolic, and maslinic acid being the predominant compounds in different HR genotypes [48,49,50]. Our research proposes that HR leaves contain up to 25-fold greater amounts of triterpenes, compared to literature data on fruits. Sadowska et al., 2020 [44], reported oleanolic and ursolic acid as the predominant compounds in the leaves of HR; however, no quantitative profiles were presented. Scientific data suggests the anticancer potential of the triterpenic compounds and, particularly, ursolic acid. Grey at al., 2010 [11], determined the antiproliferative effect of ursolic acid from HR in the Caco-2 and Hep G2 cell lines by increasing apoptosis [11]. Furthermore, the synergistic effects between the triterpenic and phenolic compounds can also potentiate the anti-inflammatory and anticancer activity mechanisms [11,44,51]. Yasukawa et al., 2009 [52], determined the anti-inflammatory and antitumor activity of HR branches and identified ursolic acid and epigallocatechin as the main contributors to the activity [52]. Skalski et al., 2018 [53], determined that sea buckthorn phenolic and triterpenic fractions are promising agents for cardiovascular diseases, as they possess anticoagulant properties and inhibit plasma lipid peroxidation.

## 3. Materials and Methods

### 3.1. Plant Material and Preparation of Extracts

The leaves of sea buckthorn (*Hippophae rhamnoides* L.), from nine different female cultivars of the selection of Botanical Garden of Moscow State University, Russia, were studied: (‘Avgustinka’, ‘Botaniceskaja Liubitelskaja’, ‘Botaniceskaja’, ‘Hibrid Percika’, ‘Nivelena’, ‘Otradnaja’, ‘Podarok Sadu’, ‘Trofimovskaja’, and ‘Vorobjovskaja’); ‘Julia’ was released in Sweden [54]. Leaf samples were collected at the Lithuanian Research Centre for Agriculture and Forestry, Institute of Horticulture (55.08911, 23.81653), in mid-August, during the phenological development stage (BBCH) 87 [55]. Leaf samples were dried using two different drying methods: convection at 60 °C and freeze-drying. Freeze-drying was performed in a Zirbus lyophilizer (Zirbus Technology GmbH, Bad Grund, Germany) at 0.01 Mbar pressure and −85 °C condenser temperature. Convection drying was performed in a UDS-150/1 hot-air laboratory dryer (“Utenos krosnys”, Lithuania) at a temperature of 60 ± 1 °C and an air-flow rate of 1.5 m s^−1^.

The dried leaves were ground in a laboratory mill Retsch ZM 200 (Retsch GmbH, Haan, Germany) using 0.2 mm ring sieve to powder and stored in tightly closed glass containers in a dark place.

For the analysis of phenolic compounds and antioxidant activity, about 0.2 g (precise weight) of HR leaf powder was weighted, and 20 mL of 70% (*v/v*) ethanol was added. For the analysis of triterpenic compounds, 1 g (precise weight) of HR leaf powder was weighted, and 10 mL of methanol was added. The extraction process continued for 15 min in an ultrasonic bath (Elmasonic P, Singen, Germany). The extracts were then centrifuged for 30 min at 3000× g in a Biofuge Stratos centrifuge and filtered through 0.22 µm pore size PVDF membrane filters (Carl Roth GmbH, Karlsruhe, Germany) to the dark glass vials. For the analysis of chlorophylls and carotenoids, about 500 mg (precise weight) of convection-dried and 200 mg (precise weight) of freeze-dried plant leaf samples were transferred to a ceramic pestle, and for the sample rehydration, 3 and 1.5 mL of ultrapure water (according to the weight of the sample) was added. The pestle was covered with aluminum foil for 2 min. The rehydrated sample was ground in a mortar and pestle with 5 g of pure quartz sand. The pigments were extracted and transferred to volumetric flask (100 mL) with an aqueous 80% solution of acetone. Homogenized sample mixture was centrifuged at 10,000 rpm for 15 min at 4 °C. The supernatant was separated and immediately subjected to analysis.

### 3.2. Chemicals

HPLC-grade chemicals and solvents were used for this study: acetonitrile, methanol, acetic, hydrochloric, trifluoracetic acids, α-amyrin, β-amyrin, β-sitosterol, lupeol, erythrodiol, maslinic acid, oleanolic acid, rutin, isoquercitrin, quercetin, isorhamnetin, procyanidin B3, caffeic acid, *p*-coumaric acid, ferulic acid, gallic acid, protocatechuic acid, caftaric acid, ellagic acid, isorhamnetin-3-rutinoside, quercetin, kaempferol, tiliroside, epigallocatechin, isorhamnetin, myricetin, quercetin 3-*O*-(6′’-acetyl-glucoside), epicatechin gallate, and resveratrol from Sigma-Aldrich (Steinheim, Germany); catechin, from Fluka (Buchs, Switzerland); uvaol, friedelin, betulin, betulinic acid, corosolic acid, rutin, isorhamnetin-3-*O*-glucoside, and quercitrin from Extrasynthese (Genay, France); ursolic acid from Carl Roth (Karlsruhe, Germany); ethanol 96% (*v/v*) (AB Vilniaus degtine, Vilnius, Lithuania); 2,2′-azino-bis(3-ethylbenzothiazoline-6-sulfonic acid) diammonium salt (ABTS), 2,4,6-Tri-(2-pyridyl)-S-triazine (TPTZ), ferric chloride hexahydrate (FeCl_3_ × 6 H_2_O), sodium acetate (CH_3_COONa), 3-(2-pyridyl)-5,6-bis-(4-phenyl-sulfonic acid)-1,2,4-triazine (ferrozine), obtained from Sigma-Aldrich (Buchs, Switzerland); potassium persulfate (K_2_S_2_O_8_), anhydrous ferrous chloride (FeCl_2_), and 6-hydroxy-2,5,7,8-tetramethylchroman-2-carboxylic acid (Trolox), obtained from Alfa Aesar (Karlsruhe, Germany). Ultrapure water was obtained by a Milli-Q water purification system from Millipore (Bedford, MA, USA).

### 3.3. Evaluation of Chromatic CIELAB Parameters

The color coordinates of the samples in the uniform contrast color space, CIEL*a*b*, were measured with a MiniScan XE Plus spectrophotometer (Hunter Associates Laboratory, Inc., Reston, VA, USA), as described in [56]. The parameters evaluated during reflected-color measurements were L*, a*, and b* (brightness, red, and yellow coordinates according to the CIE L*a*b* scale, respectively), and color saturation (the chroma value) was calculated (C = (a*^2^ + b*^2^)^1/2^), with a* and b* converted into hue angle (h° = arctan(b*/a*)) [57]. The values L*, a*, b*, and C* were measured in NBS units, hue angle h° was expressed in degrees from 0 to 360°. The NBS unit is a unit of the U.S. National Bureau of Standards and meets one color resolution threshold, i.e., the smallest difference in a color that can be captured by a trained human eye. Prior to each series of measurements, the spectrophotometer was calibrated with a light trap and a white standard with the following color coordinates in the XYZ color space: X = 81.3, Y = 86.2, and Z = 92.7. The value of L* indicated the ratio of white to black, the value of a* indicated the ratio of red to green, and the value of b* indicated the ratio of yellow to blue. The Δ*E* was calculated ($\Delta E=\sqrt{{\left(L_{2}^{*}-L_{1}^{*}\right)}^{2}+{\left(a_{2}^{*}-a_{1}^{*}\right)}^{2}+{\left(b_{2}^{*}-b_{1}^{*}\right)}^{2}}$). The ΔE values indicate the distance between colors of fresh and dried (in FD or CD mode) material. The ΔE values were added in Supplementary Material (Figure S4). Leaf powders of each cultivar were taken for the analysis. The color coordinates were processed by the Universal Software V.4-10.

### 3.4. Determination of Chlorophyll A, Chlorophyll B, and Total Carotenoid Content

The total carotenoids, chlorophyll a, and chlorophyll b content were determined spectrophotometrically, according Lichtenthaler and Buschmann [58], as described by Rubinskiene et al., 2015 [28]; the absorption was measured using a Cintra 202 spectrophotometer (GBC Scientific Equipment Pty Ltd., Australia), and the results were analyzed using the Cintral ver.2.2 program.

### 3.5. HPLC Analysis

Phenolics compounds were analyzed using the Waters e2695 Alliance system, (Waters, Milford, MA, USA), applying the method of Vilkickyte et al. [59]. Briefly, ACE Super C18 column (250 mm × 4.6 mm, particle size 3 µm; ACT, UK) was used with a gradient: 0.1% trifluoroacetic acid in water (A) and acetonitrile (B), 0 min, 15% B; 0–30 min, 30% B; 30–50 min, 60% B; 50–56 min, 90% B; 56–65 min, 15% B; the flow rate was 0.5 mL/min, injection volume −10 µL, and column temperature −15 °C. Detection of phenolic compounds was performed at a wavelength of 330, 280, and 360 nm for the phenolic acids, flavan-3-ols, and flavonols, respectively. The maximum absorption and the retention times were compared with standard compounds.

Triterpenic compounds were analyzed using the Waters e2695 Alliance system, (Waters, Milford, MA, USA), applying the methods of Vilkickyte et al. [60]. ACE C18 (150 × 4.6 mm, 3 µm) column (ACT, Aberdeen, UK) column was used and the injection volume was 10 µL. Maslinic, corosolic, betulinic, oleanolic, ursolic acids, betulin, erythrodiol, and uvaol were analyzed using the mobile phase of acetonitrile and water (89:11, *v/v*), the flow rate was 0.7 mL/min in the isocratic mode. The column temperature was set at 20 °C. Lupeol, β-amyrin, α-amyrin, friedelin, and β-sitosterol were analyzed using the mobile phase of acetonitrile and methanol (10:90, *v*/*v*). The column temperature was set at 35 °C, the flow rate was 1 mL/min. Detection of all triterpenoids was performed at a wavelength of 205 nm, corresponding to the maximum absorption and retention times, compared to standard compounds.

The obtained chromatograms have been included in the Supplementary Material.

### 3.6. Antioxidant Activity Assays

The ABTS assay was performed, as described by Re et al., 1999 [61], with some modifications, according to Raudone et al. [62]. The ferric reducing activity (FRAP) was determined, according to the method of Benzie and Strain (1996) [63], with some modifications, according to Raudone et al. [62]. All antioxidant activity measurements and calculations were performed using Trolox calibration curves and were expressed as µmol of the Trolox equivalent (TE) per one gram of dry weight, according to our previous research [62].

### 3.7. Statistical Analysis

All experiments were performed in triplicate and the results were expressed as mean ± standard deviation. Significant differences between means were evaluated using ANOVA and post-hoc Tukey’s HSD multiple comparison test. Hierarchical cluster analysis was performed using squared Euclidean distances. Principal component analysis (PCA) was performed upon factors with eigenvalues higher than 1. The linear regression model was analyzed to calculate determination coefficients. Correlations were assessed using Pearson’s correlation coefficients. Graphical and statistical analysis was performed using Microsoft Office Excel 2010 (Microsoft, JAV) and SPSS 20 software packages. The significance level was *p* < 0.05.

## 4. Conclusions

*Hippophae rhamnoides* leaves are still an underutilized resource of functional ingredients with notable antioxidant activity and rich phytochemical composition. The valorization of *Hippophae rhamnoides* leaves could conform to the strategy to transform agrotechnologcial waste into a valuables resource. Catechin, epigallocatechin, procyanidin B3, ursolic acid, α-amyrin, and β-sitosterol could be used as quantitative markers in the phenolic and triterpenic profile. The freeze-drying ensures the retainment of antioxidative active compounds, as well as notable radical scavenging and a reduction in the activities of leaf powders. The cultivars ‘Avgustinka’, ‘Nivelena’, and ‘Botaniceskaja’ were superior to other tested cultivars, with the greatest amounts of phenolic, triterpenic, carotenoid compounds, and content of total chlorophyll, as well as antioxidant activity. *Hippophae rhamnoides* leaf powders with defined phytochemical composition and determined antioxidant activity are perspective candidates in the production of smart and innovative pharmaceutical or functional food ingredients.

## Figures and Tables

**Table 1:** The chromatic characteristics (L*, a*, b*, C, and h) of convection-dried (CD) and freeze-dried (FD) HR cultivars’ leaf powders.

Cultivar	Drying Method	L*	a*	b*	C	h
Avgustinka	CD	58.76 ± 0.04	−1.37 ± 0.05	19.18 ± 0.15	19.23 ± 0.14	94.08 ± 0.15
Botaniceskaja Liubitelskaja	CD	55.92 ± 0.02	−0.30 ± 0.02	17.82 ± 0.07	17.82 ± 0.07	90.98 ± 0.05
Botaniceskaja	CD	57.76 ± 0.03	−1.24 ± 0.11	19.16 ± 0.08	19.20 ± 0.08	93.70 ± 0.32
Hibrid Percika	CD	59.53 ± 0.02	−2.67 ± 0.07	23.38 ± 0.13	24.66 ± 0.11	108.54 ± 0.25
Julia	CD	56.38 ± 0.03	−1.47 ± 0.14	18.56 ± 0.24	18.62 ± 0.25	94.53 ± 0.37
Nivelena	CD	57.12 ± 0.01	−0.74 ± 0.05	18.95 ± 0.07	18.96 ± 0.08	92.24 ± 0.14
Otradnaja	CD	55.79 ± 0.02	−1.37 ± 0.02	18.47 ± 0.07	18.52 ± 0.07	94.23 ± 0.05
Podarok Sadu	CD	59.84 ± 0.03	−5.03 ± 0.05	22.49 ± 0.10	23.11 ± 0.10	103.27 ± 0.14
Trofimovskaja	CD	60.28 ± 0.03	−5.22 ± 0.01	22.49 ± 0.08	23.09 ± 0.07	103.07 ± 0.07
Vorobjovskaja	CD	56.81 ± 0.02	−7.84 ± 0.09	19.93 ± 0.12	20.11 ± 0.12	97.63 ± 0.16
Avgustinka	FD	59.71 ± 0.02	−7.14 ± 0.06	24.62 ± 0.18	25.64 ± 0.19	106.18 ± 0.10
Botaniceskaja Liubitelskaja	FD	59.10 ± 0.04	−7.61 ± 0.03	24.68 ± 0.11	25.82 ± 0.11	107.14 ± 0.02
Botaniceskaja	FD	59.94 ± 0.03	−8.17 ± 0.03	25.02 ± 0.12	26.32 ± 0.10	108.09 ± 0.12
Hibrid Percika	FD	59.02 ± 0.04	−7.84 ± 0.09	23.38 ± 0.13	24.66 ± 0.11	108.54 ± 0.25
Julia	FD	59.18 ± 0.01	−7.66 ± 0.07	23.75 ± 0.13	24.95 ± 0.13	107.89 ± 0.15
Nivelena	FD	60.25 ± 0.01	−7.56 ± 0.04	25.06 ± 0.04	26.18 ± 0.04	106.79 ± 0.07
Otradnaja	FD	60.88 ± 0.02	−7.98 ± 0.04	24.35 ± 0.09	25.63 ± 0.07	108.14 ± 0.12
Podarok Sadu	FD	59.85 ± 0.04	−6.05 ± 0.10	22.40 ± 0.11	23.21 ± 0.12	105.11 ± 0.21
Trofimovskaja	FD	61.76 ± 0.01	−7.31 ± 0.08	24.35 ± 0.16	25.43 ± 0.17	106.71 ± 0.12
Vorobjovskaja	FD	58.69 ± 0.01	−8.25 ± 0.02	23.30 ± 0.17	24.71 ± 0.17	109.49 ± 0.10

**Table 2:** Phenolic and triterpenic profiles (µg/g, dw) of freeze-dried *Hippophae* *rhamnoides* cultivars’ leaf powders.

FD	Avgustinka	Botaniceskaja	Botaniceskaja Liubitelskaja	Hibrid Percika	Julia	Nivelena	Otradnaja	Podarok Sadu	Trofimovskaja	Vorobjovskaja
Catechin	54,685.20 ± 1894.04	39,868.50 ± 1380.78	51,592.60 ± 1786.91	46,308.60 ± 1603.87	47,683.00 ± 1651.48	60,605.80 ± 2099.14	44,232.70 ± 1531.96	43,342.10 ± 1501.11	43,620.90 ± 1510.77	44,700.90 ± 1548.18
Gallic acid	462.90 ± 15,74	401.70 ± 13.62	554,1 ± 18,9	376.30 ± 12.74	460.50 ± 15.66	565.90 ± 19.30	385.80 ± 13.07	421.70 ± 14.31	404.4 ± 13.71	453.20 ± 15.40
Protocatechuic acid	3405.10 ± 117.65	2457.20 ± 84.82	2990.80 ± 103.30	2169.90 ± 74.86	3002.00 ± 103.69	1540.10 ± 53.05	2101.20 ± 72.48	2116.80 ± 73.02	2329.00 ± 80.37	2467.20 ± 85.16
Caftaric acid	245.00 ± 8.20	162.90 ± 5.36	127.90 ± 4.16	164.10 ± 5.40	130.20 ± 4.24	164.80 ± 5.43	159.10 ± 5.23	274.10 ± 9.20	244.60 ± 8.18	206.00 ± 6.85
Ellagic acid	1880.20 ± 64.83	2157.40 ± 74.43	1921.60 ± 66.26	1339.00 ± 46.08	1659.90 ± 57.20	1816.90 ± 62.64	1692.10 ± 58.31	1244.20 ± 42.80	1396.2 ± 48.06	1413.70 ± 48.67
Coumaric acid	55.20 ± 1.69	52.00 ± 1.59	50.70 ± 1.54	68.50 ± 2.13	52.70 ± 1.61	72.00 ± 2.25	45.30 ± 1.37	48.50 ± 1.47	44.5 ± 1.34	41.90 ± 1.26
Rutin	576.10 ± 19.66	343.10 ± 11.59	532.8 ± 18.16	371.40 ± 12.57	421.60 ± 14.31	386.40 ± 13.09	310.20 ± 10.45	459.20 ± 15.61	314.6 ± 10.61	360.00 ± 12.18
Isorhamnetin-3-rutinoside	585.10 ± 19.97	542.60 ± 18.50	588.30 ± 20.08	727.60 ± 24.90	640.40 ± 21.88	479.30 ± 16.31	512.30 ± 17.45	693.20 ± 23.71	671.7 ± 22.97	746.40 ± 25.56
Isorhamnetin-3-glucoside	266.40 ± 8.94	421.60 ± 14.31	287.10 ± 9.65	256.80 ± 8.61	274.30 ± 9.21	410.90 ± 13.94	301.00 ± 10.13	368.50 ± 12.47	341.5 ± 11.54	286.80 ± 9.64
Quercetin	20.40 ± 0.66	23.30 ± 0.73	22.80 ± 0.72	20.00 ± 0.66	19.70 ± 0.65	20.10 ± 0.66	19.10 ± 0.64	18.10 ± 0.62	20 00 ± 0.66	17.80 ± 0.61
Kaempferol	21.14 ± 0.68	26.74 ± 0.82	24.61 ± 0.76	20.39 ± 0.66	18.53 ± 0.63	23.82 ± 0.74	24.33 ± 0.75	20.56 ± 0.67	13.14 ± 0.55	14.24 ± 0.56
Tiliroside	540.30 ± 18.42	253.20 ± 8.48	557.5 ± 19.01	316.80 ± 10.68	321.40 ± 10.84	338.40 ± 11.43	766.00 ± 26.23	733.7 ± 25.12	308.30 ± 10.39	370.10 ± 12.53
Epigallocatechin	38,818.50 ± 1344.41	38,931.30 ± 1348.31	34,993.50 ± 1211.90	21,464.70 ± 743.25	26,194.80 ± 907.11	40,049.30 ± 1387.04	19,971.10 ± 691.51	29,104.40 ± 1007.90	24,687.20 ± 854.88	31,498.60 ± 1090.84
Isorhamnetin	25.70 ± 0.79	28.70 ± 0.87	27.40 ± 0.83	28.00 ± 0.85	26.60 ± 0.81	28.20 ± 0.85	26.80 ± 0.82	27.00 ± 0.82	25.60 ± 0.79	25.50 ± 0.78
Myricetin	45.70 ± 1.38	47.70 ± 1.45	47.30 ± 1.43	44.60 ± 1.35	45.00 ± 1.36	44.80 ± 1.35	44.50 ± 1.34	44.30 ± 1.34	45.00 ± 1.36	45.90 ± 1.39
Quercetin 3-*O*-(6′’-acetyl-glucoside)	50.30 ± 1.53	26.60 ± 0.81	42.80 ± 1.29	47.40 ± 1.44	78,1 ± 2,46	40.80 ± 1.23	16.00 ± 0.59	12.90 ± 0.55	56.80 ± 1.74	57.00 ± 1.75
Epicatechin gallate	272.60 ± 9.15	248.00 ± 830	308.1 ± 10.38	372.70 ± 12.62	137,9 ± 4,5	418.20 ± 14.19	183.10 ± 6.06	212.40 ± 7.07	246.30 ± 8.24	309.00 ± 10.41
Ferulic acid	39.90 ± 1.20	37.80 ± 1.13	36.80 ± 1.10	36.90 ± 1.11	65,6 ± 2,04	59.50 ± 1.83	41.9 ± 1.26	31.70 ± 0.95	24.00 ± 0.75	25.10 ± 0.77
Caffeic acid	62.40 ± 1.93	84.00 ± 2.66	65.4 ± 2.03	65.8 ± 2.04	50.3 ± 1.53	64.2 ± 1.99	86.00 ± 2.73	80.20 ± 2.53	64.50 ± 2.00	66.9 ± 2.08
Isoquercitrin	190.62 ± 6.32	234.51 ± 7.84	197.73 ± 6.56	121.91 ± 3.95	155.44 ± 5.11	231.82 ± 7.74	107.17 ± 3.45	182.96 ± 6.06	149.43 ± 4.90	134.84 ± 4.40
Procyanidin B3	16,813.60 ± 582.13	16,120.70 ± 558.13	13,673.50 ± 473.36	8792.90 ± 304.29	9175.7 ± 317.55	16,470.40 ± 570.25	6619.20 ± 228.99	10,077.60 ± 348.79	11,734.50 ± 406.19	13,101.30 ± 453.54
Resveratrol	86.30 ± 2.74	66.90 ± 2.08	87.50 ± 2.78	75.20 ± 2.36	74.50 ± 2.34	73.3 ± 2.30	105.30 ± 3.38	106.30 ± 3.42	75.70 ± 2.38	80.80 ± 2.55
Maslinic aicd	176.26 ± 5.82	71.23 ± 2.23	130.25 ± 4.24	87.77 ± 2.79	146.58 ± 4.8	189.84 ± 6.29	114.39 ± 3.69	69.21 ± 2.16	43.52 ± 1.31	147.60 ± 4.84
corosolic acid	234.20 ± 7.82	143.37 ± 4.69	184.00 ± 6.09	205.11 ± 6.82	263.05 ± 8.82	232.72 ± 7.77	84.84 ± 2.69	143.98 ± 4.71	117.73 ± 3.81	232,54 ± 7,77
Betulinic acid	12.71 ± 0.55	5.65 ± 0.54	5.16 ± 0.55	9.70 ± 0.53	4.61 ± 0.55	4.46 ± 0.55	3.60 ± 0.56	4.29 ± 0.55	8.29 ± 0.53	6.68 ± 0.54
Oleanolic acid	195.32 ± 6.48	112.16 ± 3.62	177.68 ± 5.87	148.91 ± 4.88	176.66 ± 5.84	110.53 ± 3.56	54.49 ± 1.67	95.69 ± 3.06	75.59 ± 2.37	148.49 ± 4.87
Ursolic acid	657.45 ± 22.47	396.00 ± 13.42	504.12 ± 17.17	451.51 ± 15.34	523.61 ± 17.84	397.63 ± 13.48	221.53 ± 7.39	355.07 ± 12.01	248.08 ± 8.30	467.80 ± 15.91
Betulin	212.45 ± 7.07	115.11 ± 3.72	107.06 ± 3.44	113.09 ± 3.65	116.16 ± 3.76	107.39 ± 3.46	49.58 ± 1.51	86.59 ± 2.75	57.36 ± 1.76	82.00 ± 2.59
Erythrodiol	101.86 ± 3.27	35.87 ± 1.08	32.17 ± 0.97	34.60 ± 1.04	74.52 ± 2.34	67.16 ± 2.09	47.33 ± 1.43	36.15 ± 1.08	63.34 ± 1.96	36.27 ± 1.09
Uvaol	207.73 ± 6.91	79.16 ± 2.49	31.07 ± 0.93	44.04 ± 1.33	174.34 ± 5.76	146.54 ± 4.80	66.66 ± 2.07	208.77 ± 6.95	116.72 ± 3.77	153.06 ± 5.02
Lupeol	131.37 ± 4.28	61.24 ± 1.89	58.74 ± 1.81	81.21 ± 2.56	106.98 ± 3.44	158.43 ± 5.21	68.15 ± 2.12	45.20 ± 1.37	21.21 ± 0.68	116.5 ± 3.77
β-Amyrin	145.94 ± 4.78	76.33 ± 2.4	97.88 ± 3.13	41.93 ± 1.26	54.61 ± 1.67	94.24 ± 3.01	20.1 ± 0.66	47.16 ± 1.43	22.57 ± 0.71	58.42 ± 1.80
β-Sitosterol	373.81 ± 12.65	235.34 ± 7.86	168.92 ± 5.57	256.44 ± 8.59	132.94 ± 4.33	204.77 ± 6.81	116.14 ± 3.75	277.28 ± 9.31	138.71 ± 4.53	283.84 ± 9.54
α-Amyrin	972.84 ± 33.40	498.06 ± 16.96	564.59 ± 19.26	548.5 ± 18.7	354.97 ± 12.00	690.14 ± 23.61	281.45 ± 9.46	556.35 ± 18.97	225.63 ± 7.53	727.49 ± 24.90
Friedelin	182.94 ± 6.05	144.53 ± 4.73	101.86 ± 3.27	253.44 ± 8.49	274.38 ± 9.21	180.63 ± 5.97	81.07 ± 2.56	113.29 ± 3.66	60.12 ± 1.85	139.03 ± 4.54

## Data Availability

All data generated during this study are included in this article.

## Supplementary Materials

The following are available online, Figure S1: Representative HPLC-PDA chromatogram (λ = 360 and 280nm) of Hippophae rhamnoides leaf powders, showing separation of phenolic compounds. Peak assignments: 1—gallic acid, 2—epigallocatechin, 3— protocatechuic acid acid, 4—procyanidin B3, 5—caftaric acid, 6—(+)-catechin, 7—caffeic acid, 8—rutin, 9— isoquercitrin, 10—ellagic acid, 11—isorhamnetin-3-rutinoside, 12—p-coumaric acid, 13—(–)-epicatechin gallate, 14—ferulic acid, 15—isorhamnetin-3-glucoside, 16—quercetin-3-O-(6′’-acetylglucoside), 17—myricetin, 18—tiliroside, 19—resveratrol, 20—quercetin, 21—kaempferol, 22—isorhamnetin., Figure S2: Representative HPLC-PDA chromatogram (λ = 205 nm) of Hippophae rhamnoides leaf powders, showing separation of: 1—maslinic acid, 2—corosolic acid, 3—betulinic acid, 4—oleanolic acid, 5—ursolic acid, 6—betulin, 7—erythrodiol, 8—uvaol., Figure S3: Representative HPLC-PDA chromatogram (λ = 205 nm) of Hippophae rhamnoides leaf powders, showing separation of: 1—lupeol, 2—β-amyrin, 3—β-sitosterol, 4—α-amyrin, 5—friedelin., Figure S4: The difference between color of fresh Hippophae rhamnoides and dried (ΔE), using freeze-drying (FD) and convection-drying (CD).

## Sample Availability

The tested samples are available from the authors.
