# Supplementary material for: Sea Buckthorn Leaf Powders: The Impact of Cultivar and Drying Mode on Antioxidant, Phytochemical, and Chromatic Profile of Valuable Resource
Source: Molecules. 2021 Aug 6;26(16):4765. doi: 10.3390/molecules26164765 (PMC8398517; doi:10.3390/molecules26164765)
Supplement: Supplementary file 1 [file molecules-26-04765-s001.zip › molecules-1316772-supplementary.pdf]

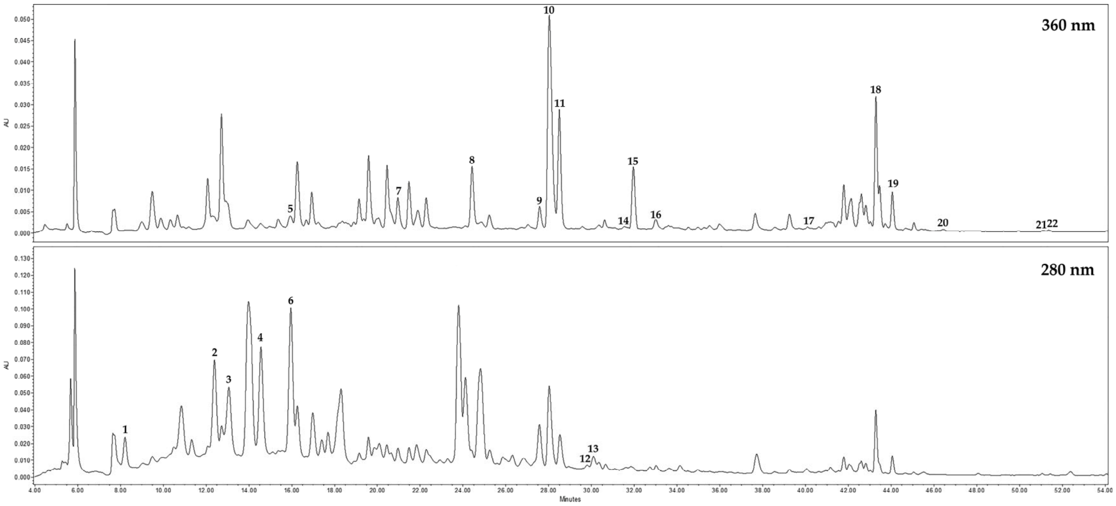

Figure S1. Representative HPLC-PDA chromatogram (λ = 360 and 280nm) of *Hippophae rhamnoides* leaf powders, showing separation of phenolic compounds. Peak assignments: 1—gallic acid, 2—epigallocatechin, 3—protocatechuic acid acid, 4—procyanidin B3, 5—caftaric acid, 6—(+)-catechin, 7—caffeic acid, 8—rutin, 9—isoquercitrin, 10—ellagic acid, 11—isorhamnetin-3-rutinoside, 12—*p*-coumaric acid, 13—(–)-epicatechin gallate, 14—ferulic acid, 15—isorhamnetin-3-glucoside, 16—quercetin-3-*O*-(6''-acetylglucoside), 17—myricetin, 18—tiliroside, 19—resveratrol, 20—quercetin, 21—kaempferol, 22—isorhamnetin.

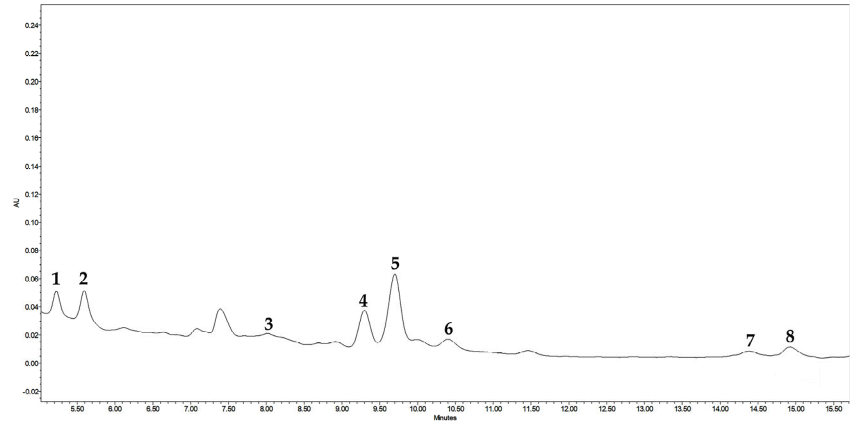

Figure S2. Representative HPLC-PDA chromatogram (λ = 205 nm) of *Hippophae rhamnoides* leaf powders, showing separation of: 1—maslinic acid, 2—corosolic acid, 3—betulinic acid, 4—oleanolic acid, 5—ursolic acid, 6—betulin, 7—erythrodiol, 8—uvaol.

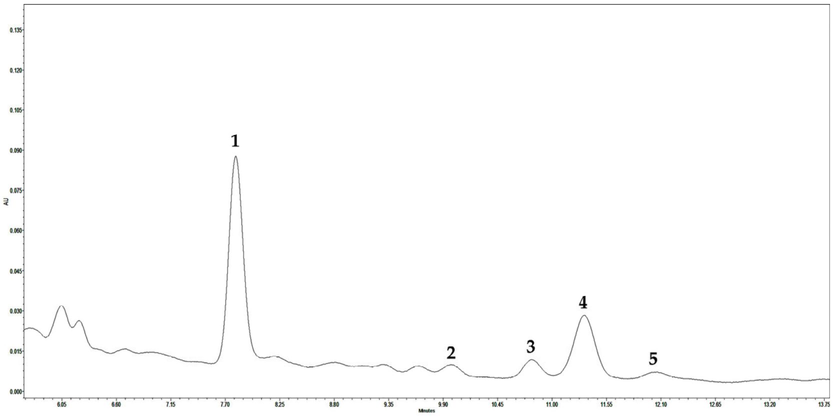

Figure S3. Representative HPLC-PDA chromatogram (λ = 205 nm) of *Hippophae rhamnoides* leaf powders, showing separation of: 1—lupeol, 2—β-amyrin, 3—β-sitosterol, 4—α-amyrin, 5—friedelin.

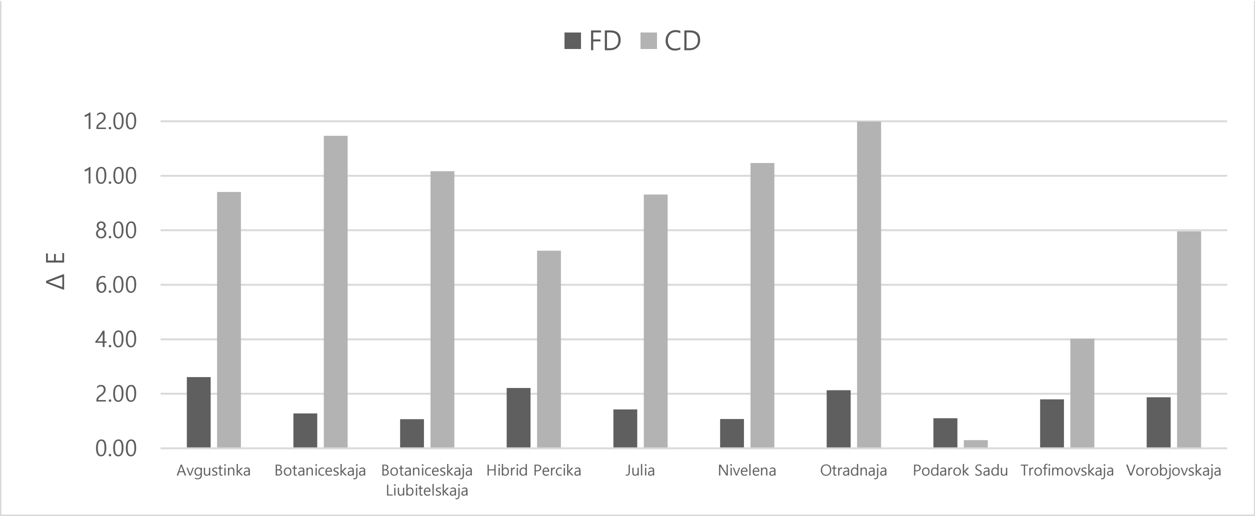

Figure S4. The difference between color of fresh *Hippophae rhamnoides* and dried (ΔE), using freeze-drying (FD) and convection-drying (CD).
